# Supplementary material for: The Abundant Distribution and Duplication of SARS-CoV-2 in the Cerebrum and Lungs Promote a High Mortality Rate in Transgenic hACE2-C57 Mice
Source: Int J Mol Sci. 2024 Jan 13;25(2):997. doi: 10.3390/ijms25020997 (PMC10815841; doi:10.3390/ijms25020997)
Supplement: Supplementary file 1 [file ijms-25-00997-s001.zip › ijms-2762756-supplementary.pdf]

Supplementary Material

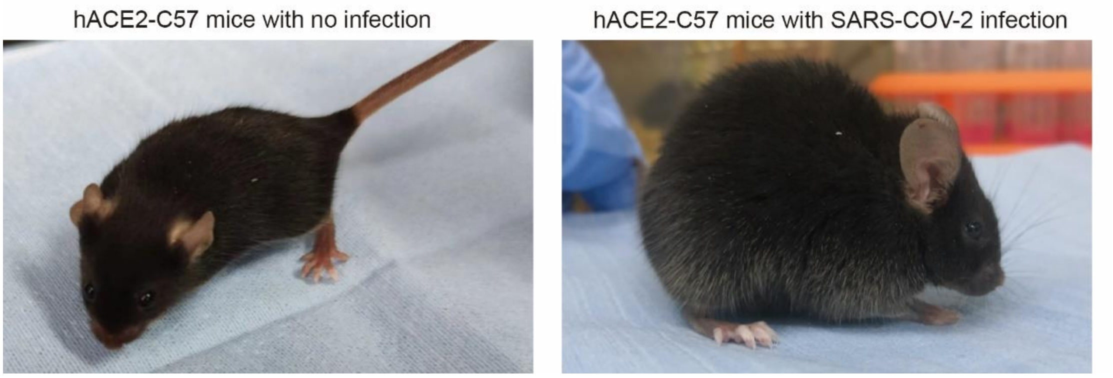

Figure S1. Clinical observation of SARS-CoV-2 infection in hACE2-C57 mice.

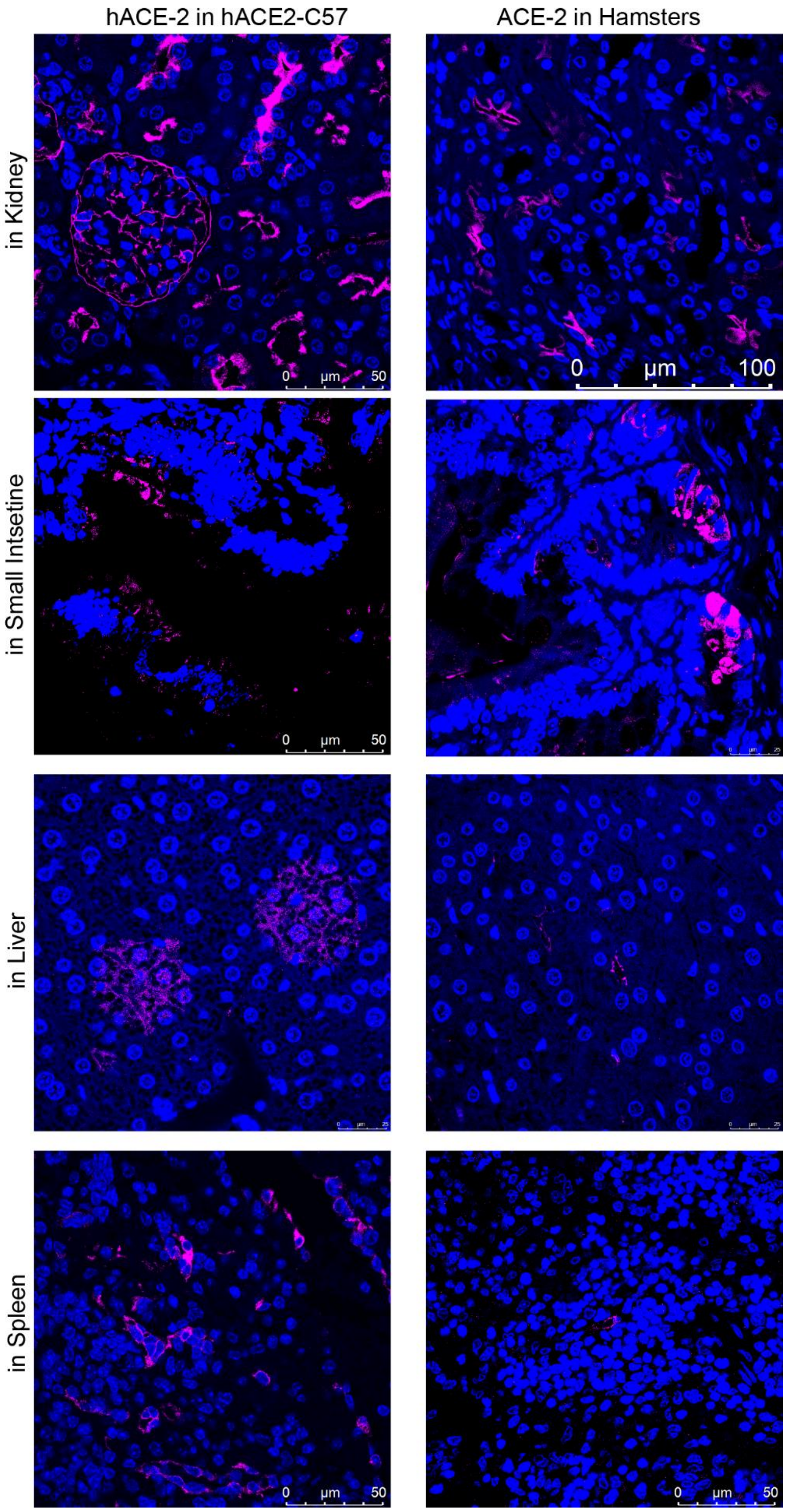

Figure S2. ACE2 distribution in the internal organs of hACE2-C57 mice and hamsters.
